# Correlation between sugar-sweetened beverage consumption and obesity among adults in Saudi Arabia

**DOI:** 10.3389/fnut.2026.1809303

**Published:** 2026-05-22

**Authors:** Faisal Saeed Al-Qahtani, Nasser F. BinDhim, Nora A. Althumiri

**Affiliations:** 1 Department of Family and Community Medicine, College of Medicine, King Khalid University, Abha, Saudi Arabia; 2 Informed Decision Making (IDM), Riyadh, Saudi Arabia

**Keywords:** body mass index (BMI), obesity, obesity risk factors, overweight, sugar-sweetened beverage consumption

## Abstract

**Background:**

In Saudi Arabia, obesity represents a major population health challenge, driven largely by unhealthy lifestyle behaviors, including excessive intake of sugar-sweetened beverages (SSBs). Nevertheless, there is a lack of evidence on the interactions between different categories of beverages and lifestyle and behavior factors that will determine the risk of obesity.

**Methods:**

This study used secondary data from the 2021 Sharik Diet and Health National Survey, a nationally representative cross-sectional phone survey in Saudi Arabia. A quota sampling approach stratified by age, sex, and region was applied. Data from 6,051 adults aged ≥18 years were analyzed, excluding participants without steady income or with extreme BMI values (<15 or ≥50 kg/m^2^). Weekly soft drink and energy drink consumption, sociodemographic characteristics, physical activity (WHO criteria), and BMI were assessed. Descriptive statistics, chi-square tests, and multivariable logistic regression were used to identify predictors of weekly beverage consumption, with significance set at *p* < 0.05.

**Results:**

Among the 6,051 participants, 38.1% were overweight and 8.3% were obese. The intake of soft drinks had a dose–response relationship with obesity with higher odds of obesity among the moderate and high consumers. It had a negative relationship with obesity with natural juice intake but no significant relationship was found with canned juices and energy drinks. The age range of 38 to 47 years showed the highest risk of obesity and males were less likely to be obese as compared to females.

**Conclusion:**

Obesity in Saudi adults is positively related to the regular use of soft-drink, but it seems that the intake of natural juice is protective. These results justify the use of public-health programs to minimize the consumption of SSBs, encourage the use of healthier beverages and customize interventions that address obesity-prevention according to age and gender. Improved research in future should draw a clear line between 100 percent fruit juices and sugar-sweetened juice drinks.

## 1 Introduction

Obesity is an extremely growing problem in the world, which is causing significant morbidity, mortality, and spending on healthcare (1). World Health Organization recognizes that obesity is a multifactorial disorder affected by behavioral, biological, environmental, and socioeconomic factors (2). Over the past few decades, the Middle East, and Saudi Arabia specifically, has been undergoing one of the most rapid obesity outbreaks globally (3). This increase can be explained by the modernization of the style of life, a lack of physical activity, the growing popularity of sedentary lifestyles, high-energy nutrition, and the ubiquity of ultra-processed products and drinks (4). Among them, the sugar-sweetened beverages (SSBs) have become one of the most powerful risk factors in dieting due to their high sugar content, quick absorption, and low satiety level (5).

SSBs are highly consumed in Saudi Arabia such as the carbonated soft drinks, energy drinks, canned juices, and sweetened natural juices (6). According to recent surveys, the consumption of soft-drinks and energy drinks is still high across many other regions and this is particularly true to the youth adults (7). Such drinks add significant levels of added sugar, mainly sucrose and high-fructose corn syrup that increase the hepatic lipogenesis, insulin resistance, visceral adiposity, and weight gain in the long term (8). In contrast to solid food, liquid calories do not trigger satiety, do not suppress the further consumption of energy, and thus enhance a chronic positive energy balance (9).

Nevertheless, the consumption of SSBs is not the cause of obesity. Dietary patterns are greatly affected by a large variety of behavioral elements, including improper timing of meals, excessive number of snacks, emotional eating, addiction to fast-food and inability to control the amount of food (10). The expansion of weight by way of diminishing the daily energy expenditure by means of physical inactivity, which is one of the most common health problems in the region, further exacerbates the problem (11). Besides this, there are psychological causes (anxiety, depression, eating under the influence of stress and the loss of control) which are also becoming listed as part and parcel of the etiology of obesity (12). The combination of the SSB consumption and these lifestyle habits provides a complex metabolic and behavioral setting that is responsive to obesity (13).

Bariatric surgeries have also been on the rise in Saudi Arabia as a clinical reaction in response to the extreme obesity and obesity related complications (14). The weight loss observed in post-surgery patient is usually dramatic, but to reach the desired long-term remission, dietary behaviors such as high-calorie drinks have to be strictly controlled (15). Although the clinical guidelines do not recommend the intake of SSBs post-bariatric surgery, a large number of patients still drink sweetened beverages, which may negatively affect the long-term weight maintenance. This group of people thus presents a highly sensitive demographic group to consider on the determinants of obesity associated with beverages (16).

Even though in the previous body of research throughout the world, SSB consumption is always linked with the weight gain, there are few region-specific studies, especially on the difference between the particular type of beverages consumed and the relationship with the lifestyle, medical, and psychological factors (17, 18). The available literature has a common tendency to group all sugary beverages together, and this might hide the significant differences between soft drinks, energy drinks, canned juices, and naturally prepared juices, as each of them differs in sugar concentration, metabolic impact, and cultural acceptance (19).

The current study aims to address these gaps by analyzing the relationship between various types of sugar-sweetened drinks and obesity and elaborating on the behavioral, medical, and activity-associated risk factors based on a large sample of Saudi adults. The study is based on the descriptive profiling, correlation analysis, and the use of logistic regression modeling that can be used in-depth to assess the contribution of beverage consumption patterns toward obesity and the interaction between the consumption patterns and the lifestyle and metabolic variables. These findings will be used to inform the health interventions of the people, clarify clinical counseling methods, and also make contributions to regional policy debates on the regulation of SSBs and the prevention of obesity.

## 2 Materials and methods

### 2.1 Study design and sampling

This study utilized a secondary analysis of Sharik Diet and Health National Survey 2021 (SDHNS). The SDHNS is a cross-sectional survey of Saudi Arabia that is conducted on an annual basis and is done via phone interviews. In order to provide proportional sample, a quota sampling technique stratified based on age group, gender, and 13 administrative regions of Saudi Arabia was used. ZDataCloud was used to manage data collection in order to reduce sampling bias. The data consisted of 6,051 adults with ages equal and above 18 years. The subjects who had no steady monthly earnings or had a body mass index under < 15 kg/m^2^ or ≥ 50 kg/m^2^ were not included.

### 2.2 Study population

All the participants were Saudi residents, aged 18 and above who participated in the 2021 SDHNS. The respondents who agreed to take part in the phone interviews gave demographic information, beverage-related information, physical activity, and self-reported height and weight. Those who do not make a consistent monthly income and those that had extreme values of BMI were not included.

### 2.3 Measurements

Individual questions were used to determine the consumption of soft drinks and energy drinks once per week: How many times per week do you consume soft drinks? And How many times per week do you consume energy drinks? The respondents answered between none and seven times per week with responses being categorized as none (0) and at least once per week (1). Sociodemographic variables included age, sex, highest educational attainment, and monthly income (categorized as < SR 5,000; SR 5,000–16,000; > SR 16,000). Body mass index (BMI) was computed from self-reported height and weight and categorized as underweight (< 18.5 kg/m^2^), normal weight (18.5–24.9 kg/m^2^), overweight (25–29.9 kg/m^2^), or obesity (≥ 30 kg/m^2^). Extreme BMI values (< 15 kg/m^2^ or ≥ 50 kg/m^2^) were excluded. The categories of physical activity were determined according to World Health Organization criteria: none, less than recommended, or meeting recommended amounts of time: 30 min or more of moderate-intensity physical activity or 20 min or more of high-intensity physical activity per week.

Beverage intake was measured by self-reported weekly intake items that were part of the SDHNS survey. The participants were questioned about the frequency of taking certain types of beverages in terms of times in a week, which included soft drinks, energy drinks, canned juice and natural juice.

Natural juice in the survey tool meant freshly made fruit juice usually served at home or bought in juice sellers and this is usually the one that the participants view this as a non-carbonated beverage that is of fruit origin. The survey however failed to explicitly differentiate between 100% fruit juice and fruit juices that have had sugar added and thus, some level of misclassification could not be ruled out.

The category canned juice implied trade packed fruit drinks, and it might consist of 100% fruit juice, as well as sweetened fruit beverages. Since these products vary significantly in the level of sugar content, this group is a heterogeneous product group of beverages.

The frequency of beverage consumption was chosen according to the days of consumption per week that were reported. There were categories of no consumption (0 days/week), low consumption (1–2 days/week), moderate consumption (3–4 days/week), and high consumption (5–7 days/week). These are the common categories of frequencies that are used in epidemiological surveys which determine the pattern of dietary intake.

### 2.4 Statistical analyses

Descriptive statistics were computed, but the summary of categorical variables was done in terms of frequencies and percentages. Chi-square tests, as a part of bivariate analyses, evaluated associations between the number of beverages with week (soft drinks or energy drinks) and the characteristics of the participants. Bivariate analyses statistically significant (*p* < 0.05) variables were put into multivariate logistic regression to define the independent predictors and determine odds ratios of weekly consumption. Adjusted models were determined to have a significant level of *p* < 0.05.

Besides the main analysis that evaluated the relationship between beverage consumption and associated obesity, a secondary logistic regression was performed to determine factors that were related to high consumption of soft-drink (3 days a week and more). This was analyzed in order to gain a better insight into the number of behavioral and demographic predictors of the high-frequency intake of soft-drinks among the population.

The multivariate logistic regression analyses were adjusted by major demographic and behavioral factors such as age, gender, monthly income and physical activity levels that were chosen as possible confounding variables based on previous literature analyzing determinants of obesity and consumption of sugar-sweetened beverages.

### 2.5 Ethical considerations

The Sharik Association of Research and studies approved the SDHNS (approval no. 06-2021). The phone interviews were conducted with all the participants giving consent. The anonymization of the data utilised in this secondary analysis was done to guarantee confidentiality and the guidelines of national research ethics.

## 3 Results

### 3.1 Sociodemographic characteristics of participants (*N* = 6,051)

The sample size was 6,051, and the ratio of females (50.3) and males (49.7) was almost equal. The most prevalent age group was 18–27 years (35.4%), then there was 38–47 years (26.1%), 28–37 years (16.2%), and 48–57 years (14.8%), and the age groups older than 58 years (58 years and above) were represented by smaller percentages. There was a balanced national representation as the participants were spread in all major regions with each region having an average of 7–8 percent of the sample representation. Such regions were Al-Baha (7.6%), Al-Jawf (7.7%), Northern Borders (7.6%), Riyadh (7.7%), Eastern Province (7.7%), Al-Qassim (7.7%), Al-Madinah (7.7%), Tabuk (7.7%), Jazan (7.7%), Hail (7.6%), Asir (7.8%), Makkah Region including Jeddah and Taif (7.8%), and Najran (7). All in all, the demographic structure exhibits a representative sample of mixed gender, age, geographical distribution (Table 1).

**Table 1:** Sociodemographic characteristics of participants (*N* = 6,051).

Variable	Category	*n*	%
Gender	Female	3,042	50.3
	Male	3,009	49.7
Age Group	18–27 years	2,142	35.4
	28–37 years	980	16.2
	38–47 years	1,580	26.1
	48–57 years	895	14.8
	58–67 years	313	5.2
	68–77 years	100	1.7
	78–87 years	29	0.5
	88–97 years	12	0.2
Region	Al-Baha	462	7.6
	Al-Jawf	464	7.7
	Northern Borders	460	7.6
	Riyadh	466	7.7
	Eastern Province	463	7.7
	Al-Qassim	468	7.7
	Al-Madinah	468	7.7
	Tabuk	468	7.7
	Jazan	466	7.7
	Hail	462	7.6
	Asir	470	7.8
	Makkah Region (including Jeddah & Taif)	471	7.8
	Najran	463	7.7
Income	No stable Income	1910	31.6
	Less than 5,000 SAR	1,401	23.2
	Between 5,001–8,000 SAR	771	12.7
	Between 8,001–11,000 SAR	599	9.9
	Between 11,001–13,000 SAR	416	6.9
	Between 13,001–16,000 SAR	377	6.2
	Between 16,001–20,000 SAR	318	5.3
	Above 20,000 SAR	259	4.3
Nationality	Non Saudi	345	5.7
	Saudi	5,706	94.3

### 3.2 Weight status categories of participants (prevalence of obesity)

Out of the 6,051 respondents, 44.3 percent were of normal weight, 38.1 percent were having overweight, 9.3 percent were having underweight with only 8.3 percent obese. In this way, nearly half of the cohort was classified as normal weight, but more than 46 per cent were classified as overweight or obese which is a clear sign of a huge excess-weight burden on this population (Table 2).

**Table 2:** Weight status categories of participants (prevalence of obesity).

Weight category	*n*	%
Underweight	564	9.3%
Normal weight	2,678	44.3%
Overweight	2,307	38.1%
obese	502	8.3%
Total	6,051	100%

### 3.3 Weekly consumption categories of soft drinks, energy drinks, and juices

The patterns of consumption of beverages among the participants differed significantly among the various beverages. In the responses to the soft drinks, close to 1/3 of the people who were asked indicated low consumption (1–2 days a week; 30.4%), then 28.4% indicated none, then 22.6% indicated moderate drinkers (3–4 days a week), and 18.5% indicated frequent drinkers (5–7 days a week). Energy drinks had been mostly avoided: 65.0% said they never used them, only 18.0% said they used them 12 days a week, 11.6% said they used them 34 days a week, and only 5.4% said they used them more than three days a week. The consumption of cans juice was more equally spread with 40.2 per cent of the 106 survey reporting none, 34.4 per cent low intake, 17.6 per cent moderate use, and 7.8 per cent high use. This was also the case with natural juice, with 37.3–1 percent not drinking it, 36.2–1 percent drinking it 1–2 days per week, 17.7–1 percent drinking it moderately, and 8.8–1 percent drinking it frequently (5–7 days per week). To conclude, the use of energy drinks was very poor, the consumption of cans and natural juice was moderated, and soft drinks were taken on a regular basis at least once a week by the study subjects (Table 3).

**Table 3:** Weekly consumption categories of soft drinks, energy drinks, and juices.

Beverage type	Consumption category	*n*	%
Soft drinks	No consumption (0 days)	1,721	28.4
	Low (1–2 days/week)	1,842	30.4
	Moderate (3–4 days/week)	1,366	22.6
	High (5–7 days/week)	1,122	18.5
Energy drinks	No consumption (0 days)	3,934	65.0
	Low (1–2 days/week)	1,092	18.0
	Moderate (3–4 days/week)	700	11.6
	High (5–7 days/week)	325	5.4
Canned juice	No consumption (0 days)	2,430	40.2
	Low (1–2 days/week)	2,080	34.4
	Moderate (3–4 days/week)	1,068	17.6
	High (5–7 days/week)	473	7.8
Natural juice	No consumption (0 days)	2,256	37.3
	Low (1–2 days/week)	2,189	36.2
	Moderate (3–4 days/week)	1,072	17.7
	High (5–7 days/week)	534	8.8

### 3.4 Cross-tabulation of beverage consumption categories and weight status with chi-square results

**Table 4:** Association between beverage consumption and weight status.

Beverage category	Normal	Underweight	Overweight	Obesity	Total
Natural juice					
No consumption	857	217	936	246	2,256
Low	1,011	203	816	159	2,189
Moderate	511	102	394	65	1,072
High	299	42	161	32	534
**χ**^**2**^ **= 90.504**					***p* < 0.001**
Canned juice					
No consumption	1,018	187	998	227	2,430
Low	945	206	768	161	2080
Moderate	477	119	399	73	1,068
High	238	52	142	41	473
**χ**^**2**^ **= 41.980**					***p* < 0.001**
Energy drinks					
No consumption	1,665	291	1,628	350	3,934
Low	531	127	361	73	1,092
Moderate	340	91	223	46	700
High	142	55	95	33	325
**χ**^**2**^ **= 104.437**					***p* < 0.001**
Soft drinks					
No consumption	840	120	635	126	1721
Low	834	156	709	143	1842
Moderate	588	147	523	108	1,366
High	416	141	440	125	1,122
χ^2^ = 64.254					*p* < 0.001

χ^2^: Chi-square value; Bold values indicates statistical significance *p* < 0.05.

Cross-tabulation analyses demonstrated statistically significant associations between beverage consumption categories and weight status for all beverage types (Table 4). Natural juice: The weight status differed significantly across levels of intake (*χ*^2^ = 90.504, *p* = 0.001). The most significant proportions of overweight and obese people were found in those who reported no consumption of natural-juices and increased consumption rates were normally linked to fewer instances of overweight and obesity. Canned juice: there was also a strong relationship (χ^2^ = 41.980, p = 0.001), but the trend was not as strong. The overweight and obese numbers were evenly distributed in the low-, as well as the moderate-intake groups. Energy drinks: A more significant association occurred (χ^2^ = 104.437, p = 0.001). Obese and overweight people were also hugely prevalent in non-consumers, but again the percentage of obesity increased in the high-intake group indicating that there was no linearity. Soft drinks: there also a significant correlation was found (χ^2^ = 64.254, *p* = 0.001). The percentage of obesity rose steadily with the no-consumption to high-consumption group, which shows that there was a definite dose–response link.

### 3.5 Multivariate logistic regression analysis of beverage consumption and obesity

The increased consumption of soft-drinks was strongly associated with increased chances of associated obesity after the factor of age and gender were controlled. Those who consumed soft drinks three to four days per week were nearly twice as likely to be obese (AOR = 1.357, *p* = 0.035), but those who used them five to seven days per week were even more likely to be obese (AOR = 1.890, *p* = 0.001). Conversely, the intake of natural juice was significantly linked with a reduced probability of obesity across all categories of intakes (AORs were 0.48 to 0.64, all *p* < 0.01). The consumption of energy drinks and canned juices were not found as important predictors. The age group with the greatest risk was 38–47 years (AOR = 1.671, *p* = 0.001) and males had the lowest probability of obesity (AOR = 0.788, *p* = 0.014) (Table 5).

**Table 5:** Multivariate logistic regression model examining the association between beverage consumption and obesity after adjustment for age, gender, income, and physical activity.

Variable	Category	AOR [Exp(B)]	95% CI	*p*-value
Soft drinks (Soda)	Low (1–2 days)	1.236	0.953–1.603	0.110
	Moderate (3–4 days)	**1.357**	**1.022–1.802**	**0.035**
	High (5–7 days)	**1.890**	**1.422–2.514**	**<0.001**
Energy drinks	Low	0.797	0.605–1.051	0.108
	Moderate	0.788	0.560–1.108	0.171
	High	1.194	0.790–1.803	0.400
Canned juice	Low	1.015	0.804–1.282	0.899
	Moderate	0.965	0.712–1.307	0.819
	High	1.174	0.802–1.719	0.410
Natural juice	Low	**0.639**	**0.508–0.803**	**<0.001**
	Moderate	**0.524**	**0.384–0.716**	**<0.001**
	High	**0.481**	**0.317–0.732**	**0.001**
Age group	28–37 yrs	1.018	0.759–1.363	0.907
	38–47 yrs	**1.671**	**1.270–2.198**	**<0.001**
	48–57 yrs	1.469	0.955–2.259	0.080
	58–67 yrs	1.749	0.882–3.468	0.110
	68–77 yrs	1.771	0.523–5.990	0.358
	78–87 yrs	1.496	0.189–11.836	0.703
Gender	Male	**0.788**	**0.652–0.952**	**0.014**

AOR, Adjusted Odds Ratio; CI, Confidence Interval. Multivariate logistic regression was adjusted for age, gender, income, and physical activity. Bold values indicate statistically significant associations (*p* < 0.05).

### 3.6 Multivariable logistic regression analysis predicting high soft-drink consumption

Dependent variable: High soft-drink consumption (≥3 days/week).

As a secondary analysis, a multivariable logistic regression model was conducted to identify predictors of high soft-drink consumption (≥3 days per week). Logistic Regression Analysis Predicting High Soft-Drink Consumption. After adjusting for age, gender, income, BMI category, physical activity, and eating habits, natural juice intake was not independently associated with high soft-drink consumption (OR = 1.13, *p* = 0.057). This suggests that individuals who consume less natural juice are not necessarily the same individuals who consume excessive soft drinks, although a borderline trend was observed (Table 6).

**Table 6:** Multivariable logistic regression analysis predicting high soft-drink consumption.

Variable	Category/unit	Adjusted OR (ExpB)	95% CI	*p*-value
Natural juice consumption	(per unit increase)	**1.13**	0.996–1.277	0.057
Age group	Overall	—	—	<0.001
	18–27 years (ref)	1.00	—	—
	28–37 years	3.86	1.00–14.81	0.049
	38–47 years	3.59	0.93–13.84	0.063
	48–57 years	2.67	0.69–10.27	0.153
	58–67 years	1.86	0.48–7.17	0.369
	68–77 years	1.13	0.29–4.45	0.858
	78–87 years	0.89	0.21–3.74	0.878
	88–97 years	1.07	0.22–5.29	0.937
Gender	Male vs. Female (ref)	**0.51**	0.45–0.57	<0.001
Income	Overall	—	—	0.511
BMI category	Overall	—	—	<0.001
	Ideal (ref)	1.00	—	—
	Underweight	**0.63**	0.51–0.77	<0.001
	Overweight	0.98	0.76–1.26	0.866
	Obese	0.85	0.69–1.04	0.114
Vigorous PA (VIPA)	per unit	1.00	0.96–1.03	0.857
Moderate PA (MIPA)	per unit	0.99	0.96–1.02	0.567
Number of meals/day	per unit	**1.19**	1.07–1.32	0.001
Number of snacks/day	per unit	**1.30**	1.21–1.40	<0.001

Model fit statistics: χ^2^ (23 df) = 439.73, *p* < 0.005; Nagelkerke *R*^2^ = 0.094, Hosmer–Lemeshow test *p* = 0.005; overall classification accuracy = 63.6%.

## 4 Discussion

The current research evaluated the relationship between the use of soft drinks, energy beverages, canned juices, and natural juices and concurrence of obesity in a nationally representative sample of 6,051 adult participants. Findings indicate that the beverage habits are closely related to the weight status: soft drinks have a direct dose–response correlation with obesity, whereas intake of natural juices has an inverse relationship. These results add to the ever-growing evidence that sugary sweetened drinks cause excess weight gain, and healthier drinks options can be inverse association.

The present study demonstrated a clear dose–response relationship between soft drink consumption and obesity. In the adjusted model, participants consuming soft drinks 3–4 days per week had higher odds of obesity (AOR = 1.36; 95% CI: 1.02–1.80; p = 0.035), while those consuming soft drinks 5–7 days per week had even greater odds (AOR = 1.89; 95% CI: 1.42–2.51; *p* < 0.001) compared with non-consumers (20, 21). As an example, Reppas (22) found out that the risk of obesity and diabetes is significantly increased by habitual soft-drink consumption among several cohorts (22). Another study showed the same result where those adults who took fewer than four portions of soft drinks each week were found to have a lower BMI than those who took more than four portions a week (23, 24). Furthermore, a systematic review of literature by Lundeen et al. (25) has revealed consistently positive correlations between the consumption of SSBs and BMI in high, middle, and low-income nations (25). These observations are supported by the current study and point to the unhealthy eating habits promoted by the popular access and repeated use of soft drinks. Along with investigating the relationship between beverage consumption and obesity, the current study examined demographic and behavioral variables linked with high intake of soft-drinks. Knowledge of predictors of high-frequency soft-drink consumption can be used to inform specific population-health target interventions that can be used to reduce the intake of sugar-sweetened beverages by high-risk population groups. This secondary analysis also makes more sense of behavioral trends that might lead toward overconsumption of sugar-sweetened drinks.

Regular intake of natural juice on the other hand was associated with reduced risk of obesity at all levels even after age and gender became known. The odds of being obese were half in people consuming natural juice 34 days a week and even greater in those consuming it 57 days a week (adjusted odds ratio = 0.481). Such trend can be explained by the nutrient content and fiber of fresh natural juice, which increase satiety, better glucose regulation, and substitutes energy-rich soft drinks along with these also explained by those kind of people may choose more healthier lifestyle options and consuming healthier foods as well (22). The same findings were obtained by Fardet et al. (26) who concluded that increased consumption of fruit and fruit-based drinks was correlated with lower weight gain in longitudinal cohorts (26). Another study, also established that regular consumption of natural juices was associated with reduced BMI and healthy eating behavior, though the authors stated that such an advantage depends on the lack of added sugar (27).

The consumption of energy-drink analysis showed that there was statistically significant association in the chi-square analysis but no significant relationship in the adjusted logistic-regression models. The trend indicates that the younger age and exercising among males can be the demographic or lifestyle confounders hence the lower levels of obesity risk in the unadjusted cross tabulations (28). The previous studies have given contradictory data: some of them indicate that energy-drink consumption is linked to increased caloric intake and metabolic susceptibility (29, 30). Our results are thus consistent with the perception that the use of energy drinks can lead to poor health behaviors but not necessarily leading to obesity (31, 32).

Our results revealed that there was no significant association between the intake of canned juice and obesity adjusted by the covariates this was similar to study that showed same response (33). This is probably indicative of the high range of canned juices: some of them are highly sugar-sweetened, others have minimal added sugar content, resulting in mixed nutritional values (34). The previous studies on packaged juices have given conflicting findings (34). An example of such an instance is that, in a study by Wang et al. (35) where high consumption of packaged fruit drinks was the dependent variable, higher intake of such drinks was correlated with higher BMI among the U. S. adults (35), but in a study by Apergi et al. (36), no significant relationship emerged when all sociodemographic factors were controlled and no significant association between NSS soft drink intake (36). This uncertainty is reflected in the current findings and highlights the need to identify the difference in 100% fruit juice and sugar-sweetened juice beverages in future research.

The impact of demographic factors was also significant, in particular, age and gender. Mid-adulthood was found to be an at-risk phase to increase weight, as adults aged 38–47 years had a higher probability of becoming obese compared to younger adults (adjusted odds ratio = 1.671), which is corroborated by worldwide evidence that suggests that mid-adulthood is a high-risk period due to a lower level of physical activity and metabolic changes (37). Men on the other hand were much less likely to live with obesity compared to women (adjusted odds ratio = 0.788). This trend also conforms to the results of other studies conducted in the Middle East such as explained the increased frequency of obesity among women by cultural, behavioral, and hormonal differences (38). Model diagnostics indicated some limitations in model calibration, as suggested by the Hosmer–Lemeshow goodness-of-fit test reported in the regression analysis. Although the regression models remained statistically informative, this result suggests that additional unmeasured variables may influence obesity risk. Future studies incorporating more detailed dietary intake measures and lifestyle variables may improve model calibration and predictive performance.

## 5 Conclusion

The current study shows a close correlation between beverage-drinking patterns and obesity among adults highlighting the role that food preferences play in determining the weight of the population. The relationship between soft drinks and obesity showed a clear dose–response relationship, the more one consumed, the more the chances of becoming obese. On the other hand, the use of natural juices was always linked to a reduced risk of obesity, indicating that there is a possibility of effect, in case of the absence of added sugars. Energy drinks and canned juices became insignificant following the adjustment factor which shows that their observed effect could be confounded by demographic or lifestyle variables. Age and gender were no longer irrelevant factors of obesity with the highest risk being adults aged 38–47 years.

### 5.1 Recommendations

These results underscore the necessity of reducing the consumption of sweetened drinks with sugar and promoting the consumption of healthier alternatives like natural juices and not sugar-sweetened ones as national obesity-prevention measures. These findings may be used to guide the policies of the public-health, including dietary advice and education to specific interventions that encourage improved beverage selection. With the emphasis on the changeable eating patterns, particularly, high intake of soft drinks, we can accomplish quite a lot to alleviate the increasing obesity rate in adults.

### 5.2 Study limitations

One of the constraints of this study is associated with the classification of juice drinks. The survey could classify the natural juice and canned juice as beverages that have different levels of sugar in them. Members could have understood natural juice to mean freshly made juice, however, some members could have used fruit drinks with sugar added. Likewise, the category of products such as cans of juice will probably comprise a heterogeneous blend of products that vary in composition to 100% fruit juice to sugar-sweetened fruit juices. This possible misplacing can affect the identified associations, especially the apparent protective association that has been observed in the use of natural juice. In future research, the distinction between 100 per cent. Fruit juice and sugar-sweetened fruit drink should be made to gain a clearer insight into the relative contribution of these beverages to the risk of obesity.

The other limitation of the study is that it lacks data about overall caloric intake and the general eating habits in the survey data. Another confounding factor that is significant in the study of the association between certain drinks and risk of obesity is dietary intake. The drinkers of sugary drinks might also have other diet-related habits, including an increase in the intake of calorie-rich food or fast food, which might affect body weight. These variables were not provided in the SDHNS dataset and therefore, they were not present to be incorporated in the multivariate models. Hence, the findings on the relationships between the intake of drinks and obesity cannot be considered reliable because there might still be some residual confounding that cannot be accounted by unmeasured factors in the diet.

The current research design employed a cross-sectional design, and thus, no causal relationships are possible. The reported relationships between beverage intake and obesity could also be as a result of reverse causation whereby obese individuals change their beverage consumption as weight-control behaviors. In one instance, obese people can decrease the soft drinks or drink more perceived healthier soft drinks like natural juice. Hence, the results are to be taken as associations, but not causal relationships.

## Data Availability

The data analyzed in this study is subject to the following licenses/restrictions: the Sharik Board permits data access to members and researchers following submission of a formal request and approval by the designated board. Requests to access these datasets should be directed to na@idm.sa.
